# Umbilical Endometriosis: A Case Report

**DOI:** 10.7759/cureus.97641

**Published:** 2025-11-24

**Authors:** Hamdi Al Shenawi, Sara Al Buhmaid, Fatima Al Shenawi, Muneera AlRumaihi, Nawal Alhamar

**Affiliations:** 1 Surgery, Arabian Gulf University, Manama, BHR; 2 Medicine, Arabian Gulf University, Manama, BHR; 3 Radiology, Princess Al-Jawhara Centre, Manama, BHR

**Keywords:** cutaneus endometriosis, diagnosis, extragenital endometriosis, primary umbilical endometriosis, treatment

## Abstract

Endometriosis is characterized by the ectopic presence of endometrial tissue outside the uterus. When this tissue appears in regions beyond the reproductive organs, it is referred to as extragenital endometriosis. One rare form of this condition is primary umbilical endometriosis, where ectopic endometrial tissue is found in the umbilicus. Common symptoms include an umbilical mass and cyclical discharge related to menstruation. However, diagnosis is often delayed due to its rarity. The exact cause of this condition remains unclear. While histological examination is considered the gold standard for diagnosis, imaging techniques can also play a significant role. We present a case of extragenital endometriosis of the umbilicus. The patient experienced cyclical pain and swelling in the umbilicus. A CT scan revealed a nodule in the umbilicus with no accompanying intra-abdominal pathology. Complete excision of the umbilicus confirmed the diagnosis of an umbilical endometrioma. Our goal is to highlight the importance of considering umbilical endometriosis in the differential diagnosis of abdominal or umbilical pain, especially in women of reproductive age, to improve patient outcomes.

## Introduction

Extragenital endometriosis (also called extra-pelvic endometriosis) refers to functional endometrial glands and stroma located outside the uterine cavity and muscle wall [1]. Endometriosis is classified into two types: endometriosis interna and externa. Endometriosis interna (adenomyosis) occurs when endometrial tissue grows within the myometrium. In contrast, endometriosis externa involves endometrial tissue implanted in other sites, such as pelvic regions proximal to the uterus (e.g., fallopian tubes and ovaries) or distant locations outside the pelvis, including the gastrointestinal tract, urinary tract, pulmonary system, extremities, skin, and central nervous system [2-4]. The epidemiology, pathogenesis, diagnosis, and natural history of endometriosis interna differ from those of endometriosis externa [1,3,5]. The true prevalence of extrapelvic endometriosis remains uncertain due to a lack of thorough epidemiological studies. However, it is estimated to be between 1% and 5% in women of reproductive age and approximately 13% in those who are infertile [6]. 

This was first described by Rokitansky in 1860 during an autopsy after he examined a uterine polyp, an unusual tissue growth in the uterus and discovered that it had similarities to endometrial tissue [6,7]. Endometriosis externa most commonly presents with symptoms such as infertility, dysmenorrhea, dyspareunia, and deep pelvic dull aching pain [1,3,4,5]. Several theories have been proposed to explain the pathophysiology of pelvic endometriosis. Among the most accepted theories are retrograde menstruation into the peritoneal cavity and iatrogenic implantation of endometrial tissue during pelvic procedures [5]. Primary umbilical endometriosis is rare and characterized by endometrial tissue within the umbilicus. Its pathophysiology remains unclear and shows no obvious link to seeding during surgical procedures [5,7]. In this context, we report a case of primary umbilical endometriosis.

## Case presentation

A 38-year-old married woman arrived at the surgical emergency department complaining of a painful umbilical swelling of a few hours’ duration. The pain was severe, dull aching, and not responding to oral analgesics. She used to have similar milder cyclical pain during the premenstrual period for more than two years. The patient denied having fever, loss of appetite, gastrointestinal, or urinary symptoms. The patient's medical history included a lower-segment cesarean section performed via a Pfannenstiel incision five years ago. She had been trying to conceive for the past three years, yet she had not consulted a healthcare professional.

**Figure 1:**
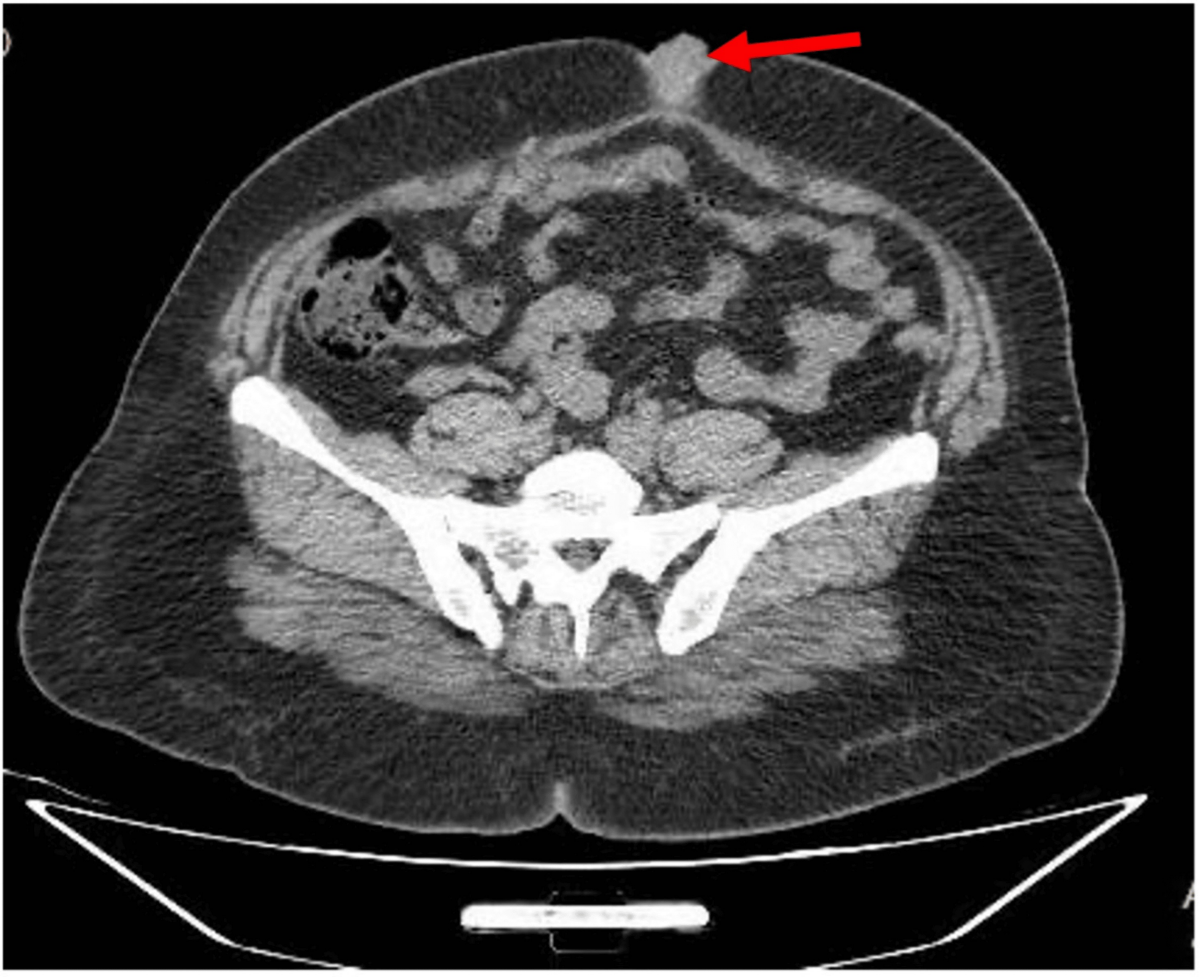
Axial reformatted images of an unenhanced CT scan of the abdomen and pelvis show an irregular soft tissue lesion in the umbilical region measuring 3.0 x 2.6 cm. The lesion is slightly hyperdense in the plain scan with no associated calcifications or cystic changes

**Figure 2:**
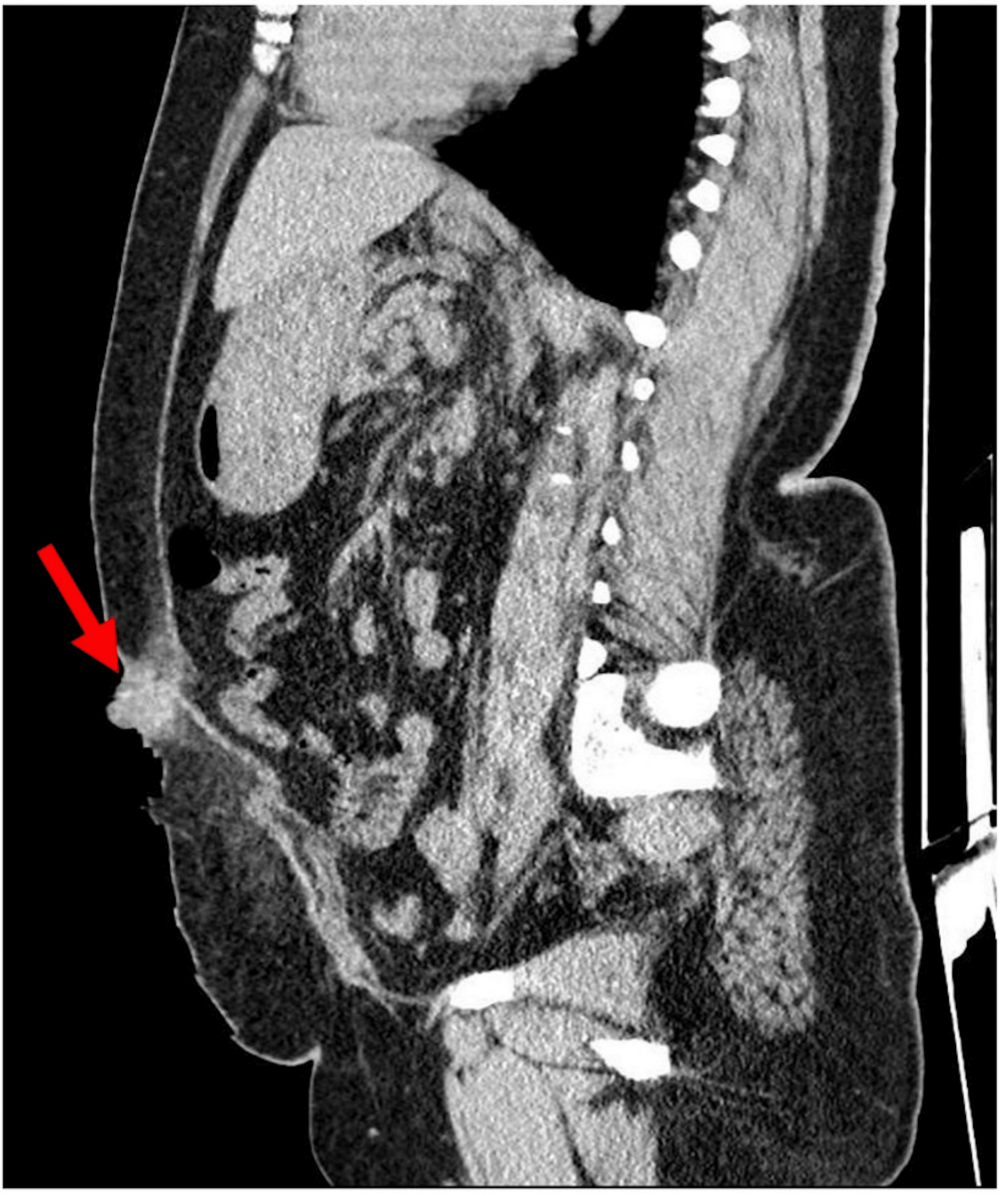
Sagittal-view CT scan revealing the umbilical lesion

Upon examination, the patient was afebrile and exhibited stable vital signs. An abdominal examination revealed an irreducible, mildly tender umbilical swelling characterized by smooth, well-defined margins and no discoloration. The swelling measured approximately 3 x 3 cm and had a partially bulging anterior profile. Chest examination was unremarkable. CT of the abdomen showed nodular soft tissue thickening at the umbilical level of the anterior abdominal wall, about 3 x 2.6 cm, and was seen partially bulging anteriorly. (Figures 1, 2). The provisional diagnosis was endometrioma of the umbilicus or, rarely, an incarcerated hernia.

**Figure 3:**
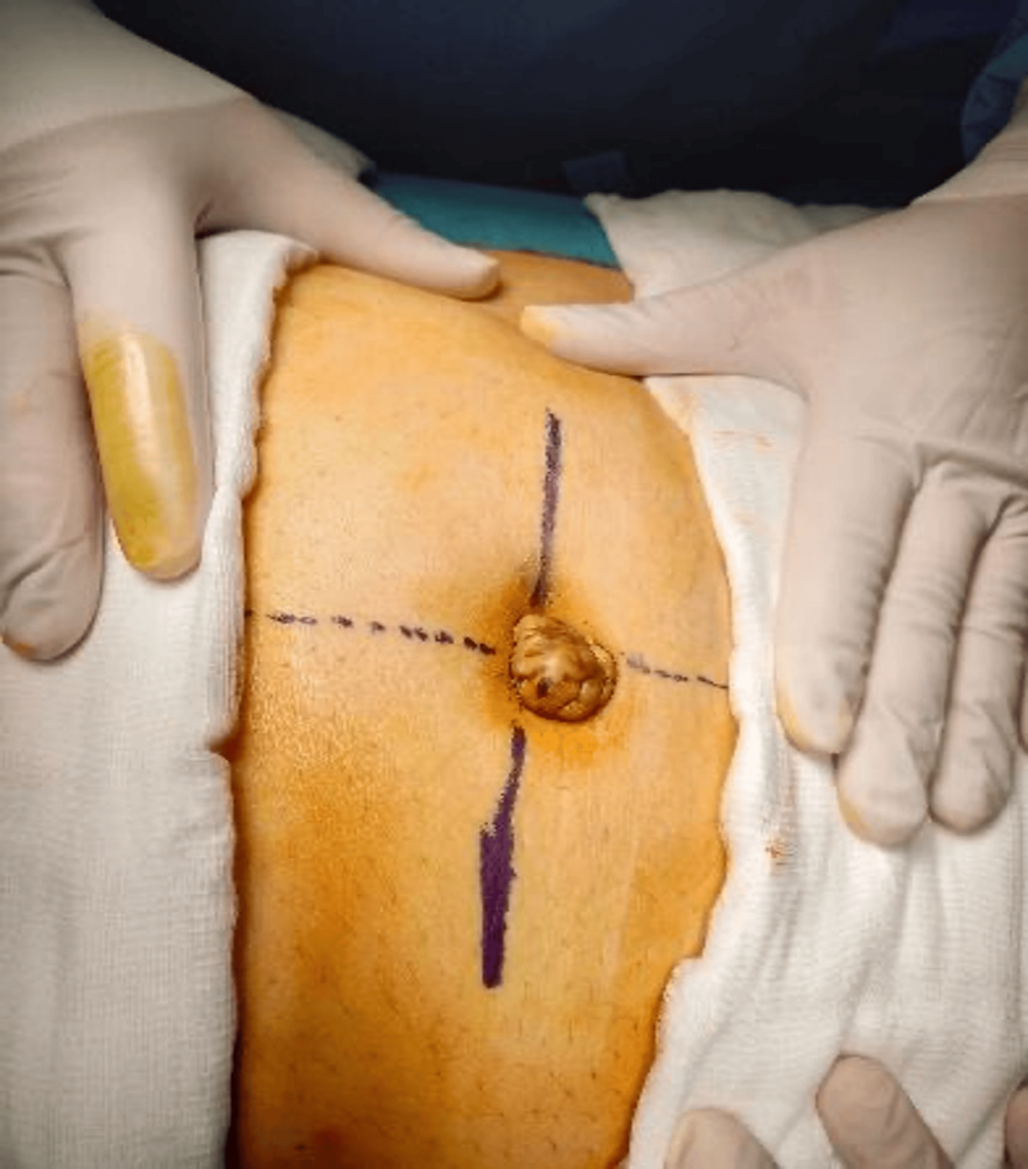
Pre-operative photo of the umbilical swelling

**Figure 4:**
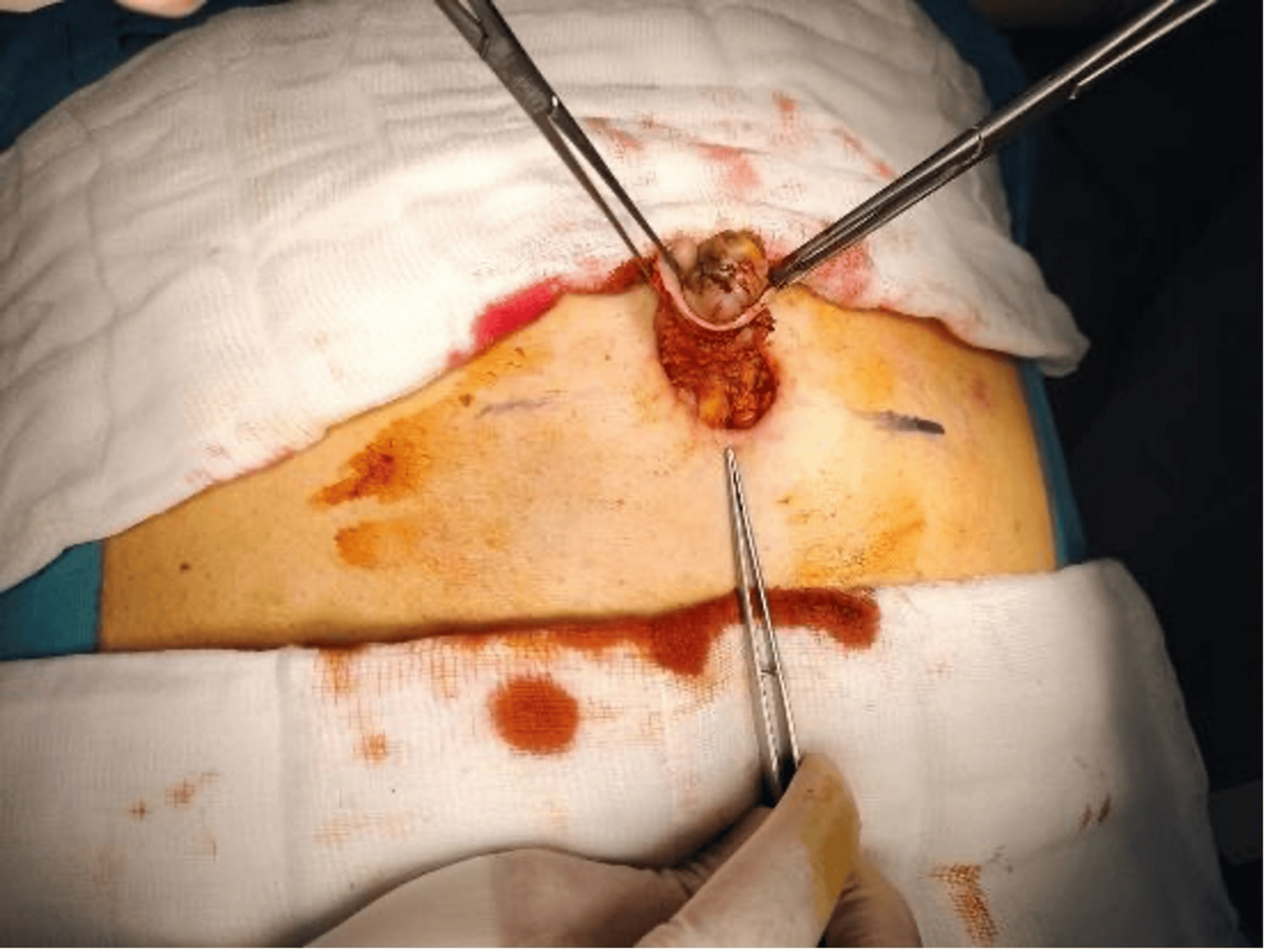
A periumbilical free margin circular incision was performed

**Figure 5:**
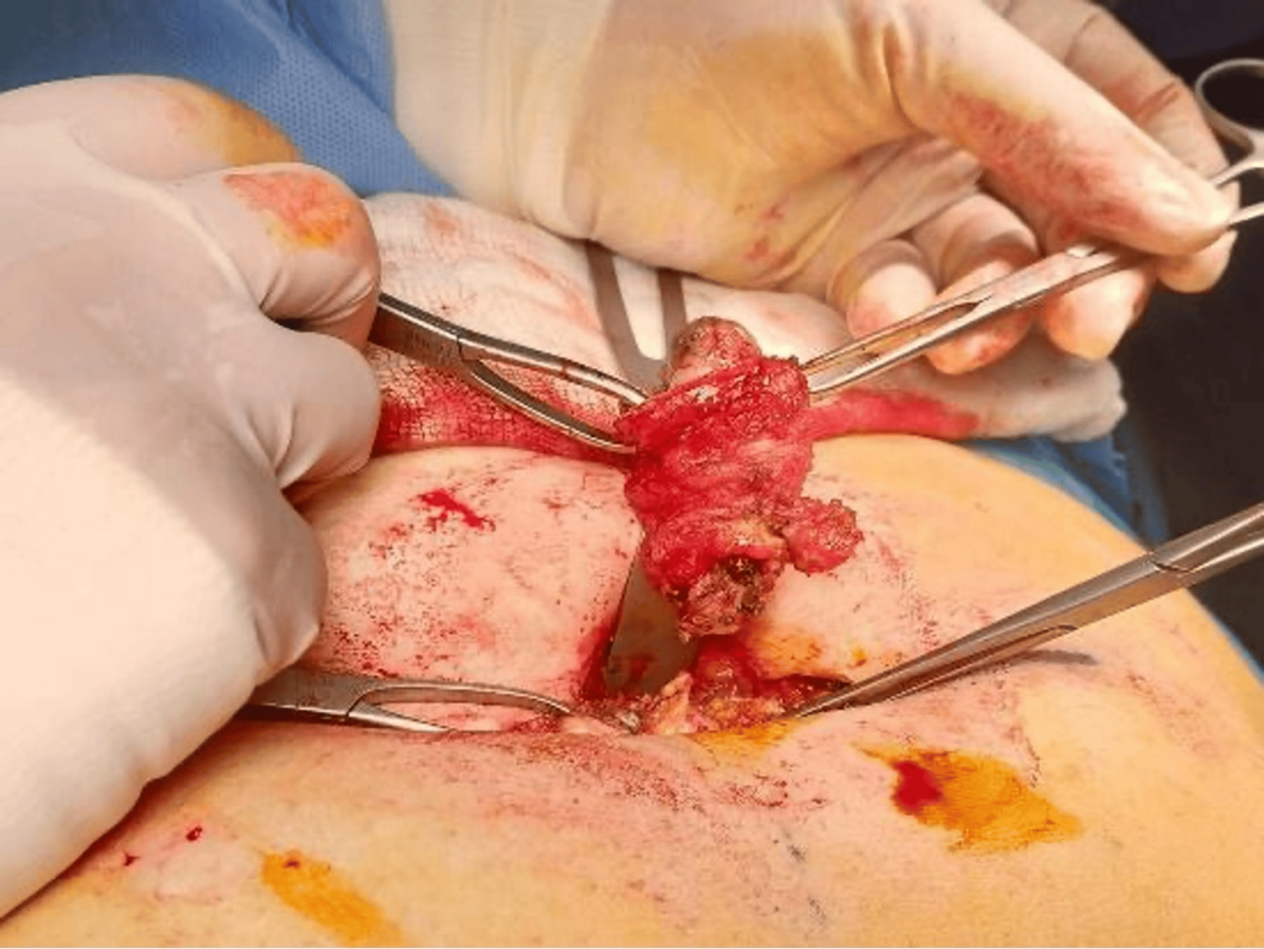
The incision is transcutaneous and completed to include the fascia and peritoneum

**Figure 6:**
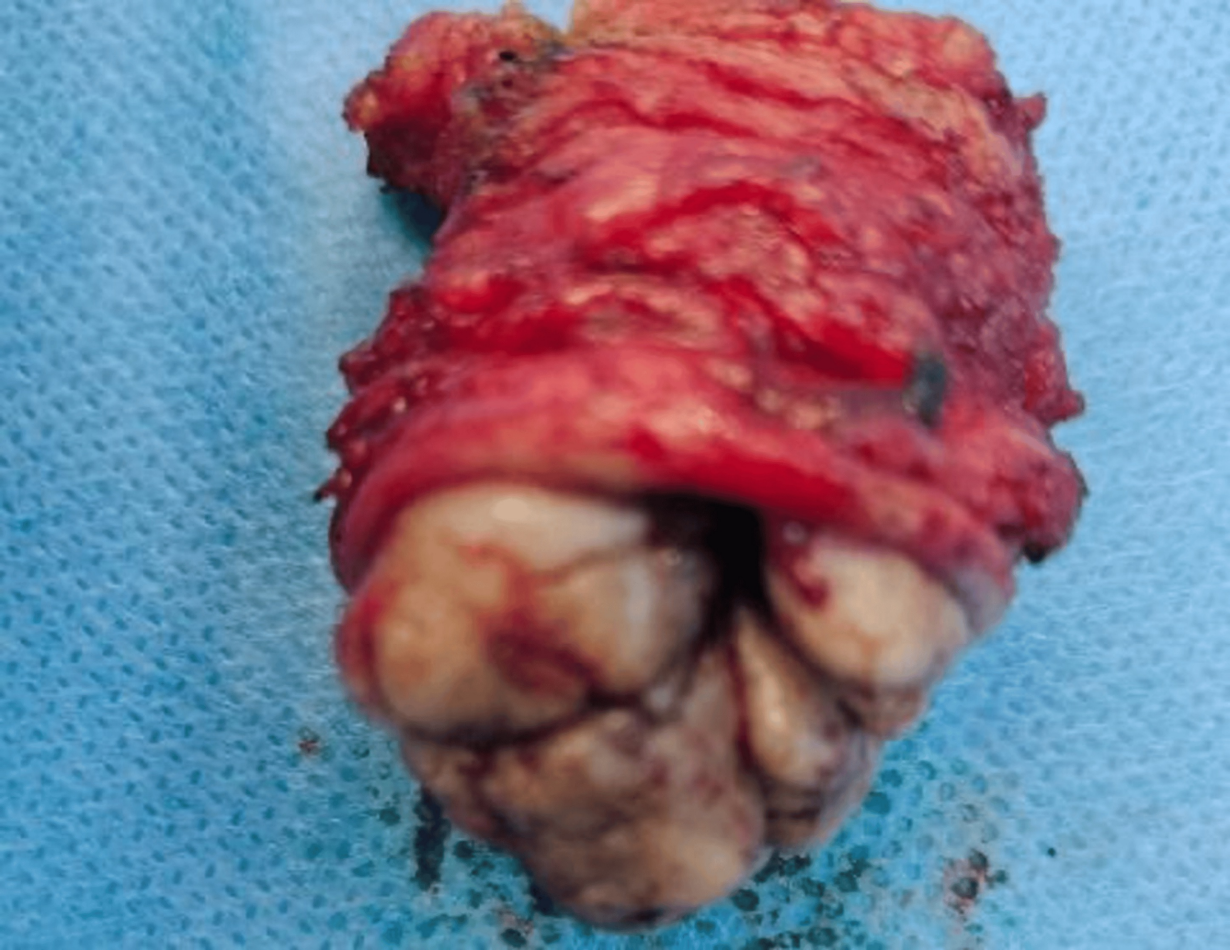
The nodule was completely excised

The next day, the patient underwent exploration with wide local excision of the umbilicus down to the peritoneum with repair of the defect and primary closure (Figures 3-6). 

The periumbilical free margin incision was performed to gain access to the underlying tissues while preserving the integrity of the surrounding structures (Figures 4, 5). It also ensures that the incision is within the free margin to minimize scarring and promote optimal healing.

Complete excision of the lesion was done, which is the gold standard treatment of periumbilical endometriosis (Figure 6). The patient recovered very well.

**Figure 7:**
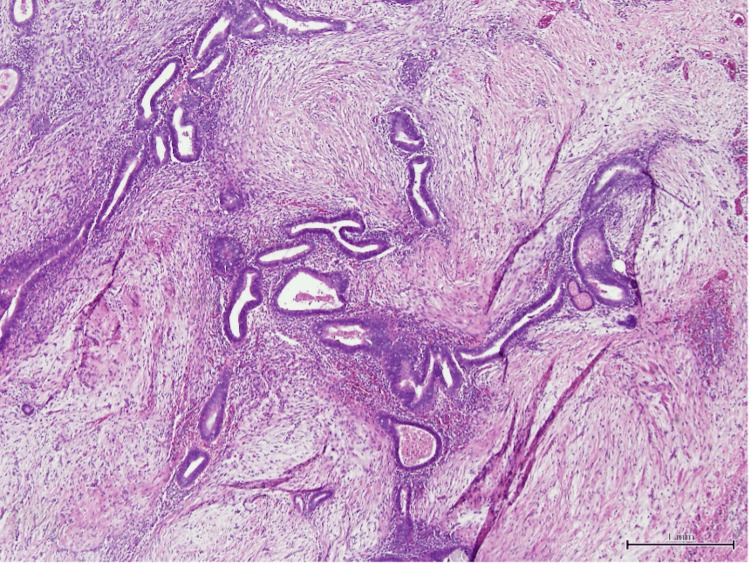
Histopathological assessment of the umbilical lesion resection showing an ectopic endometrial gland and stroma (H & E stained, original magnification X40)

**Figure 8:**
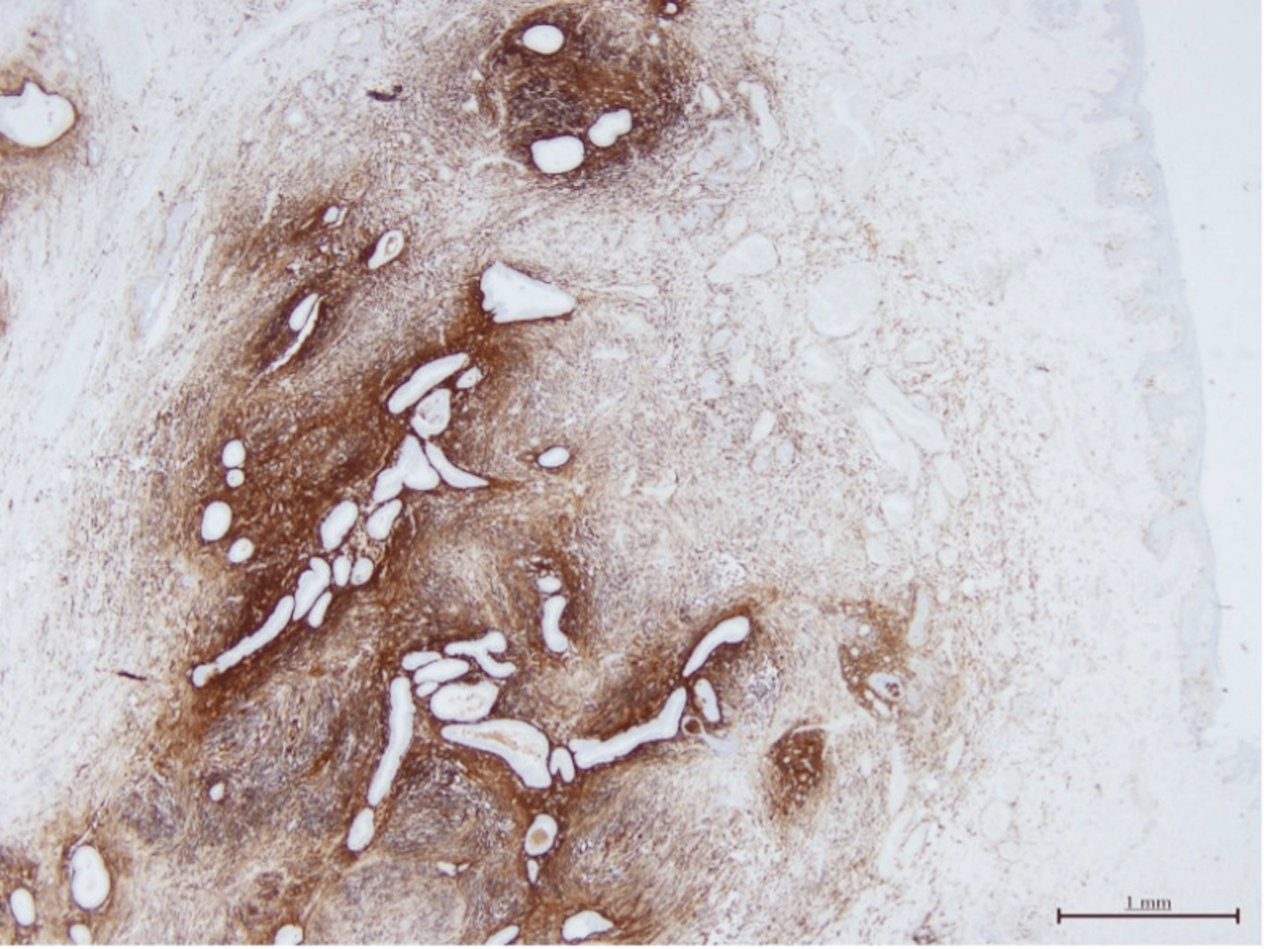
Immunohistochemistry highlighting the endometrial stroma (CD10 immunostain, original magnification X20)

Histopathology confirmed the diagnosis of endometriosis of the umbilicus, presenting with surrounding free margins. The biopsy revealed multiple foci of ectopic endometrial tissue, along with secondary stromal hemorrhage and chronic fibroinflammatory tissue reactions (Figure 7). Additionally, focal areas of cystically dilated glands with secretions and calcification were observed. No other significant abnormalities or atypia were noted. Immunohistochemistry of the lesion showed diffuse positivity for CD10 and estrogen receptors, further supporting the diagnosis of endometriosis (Figure 8).

Follow-up for two years showed no clinical or radiological recurrence.

## Discussion

Abdominal wall endometriosis is an extra-pelvic, external endometriosis. It is classified into primary (spontaneous) or secondary to pelvic interna or externa endometriosis [1,5,7]. In primary endometriosis, endometrial tissue grows in the abdominal wall outside the uterus, with no previous history of pelvic surgery or prior history of uterine endometriosis [8]. Secondary endometriosis occurs in patients who have had pelvic or laparoscopic surgeries [9], have concomitant pelvic endometriosis, like ovarian endometrioma, or sometimes have endometritis, leiomyoma of the uterus, etc. [10,11]. It is usually found superficial to the peritoneum [5,7].

The pelvic endometriosis median age of diagnosis is reported to be between 25 and 30 years, as opposed to 34 and 40 years in extrapelvic endometriosis [1]. Furthermore, the prevalence of endometriosis in women of childbearing age is estimated to be 5% to 10%, with most studies confirming that only 0.5-1.0% of these women carry the diagnosis of endometriosis in an extra-pelvic site [5-7]. The umbilicus is the most common affected site, followed by the inguinal and anterior abdominal wall, which mainly affects parous women [5,7]. Several cases have been documented, including the one by Nellihela et al. that highlighted a case of a 16-year-old girl with a history of autism and precocious puberty, who presented with a painful umbilical lump for a 2 to 3 months duration [12]. Choi et al. also presented a case of a postmenopausal woman with no past history of any surgical procedures diagnosed with spontaneous umbilical endometriosis [13], and Lugata et al. in a 35-year-old nulliparous woman [14]. Other reports highlight umbilical endometriosis coexisting with multiple fibroids or in conjunction with a bicornuate uterus [15]. One case of spontaneous umbilical endometriosis was reported in a pregnant lady without any previous surgery [16].

Patients often present with cyclic symptoms of umbilical swelling and pain, with the umbilical mass fluctuating in size according to the menstrual cycle [3-5]. During the premenstrual period, increased pain and swelling occur due to the congestion of the endometrial tissue both in the umbilicus and the uterus. This explains the cyclical symptoms of pain related to the menstrual cycle, and in some instances, bleeding from the umbilical area during menstruation [5,7]. The appearance of the umbilicus can vary, appearing fleshy, brownish, or reddish. Rachagan et al. reported a case of catamenial pneumothorax, in which lung collapse occurs during or around menstruation [17]. The patient was started initially on danazol twice daily after being diagnosed with umbilical and pulmonary endometriosis but presented two months later with recurrence of symptoms. Due to the poor response to medical therapy, she underwent a total abdominal hysterectomy and bilateral salpingo-oophorectomy along with the excision of the umbilical nodule. When followed up years later, the patient appeared to be doing well. This rare manifestation of thoracic endometriosis is challenging to diagnose and treat, with a high rate of recurrence [17]. 

Imaging plays a critical role in diagnosis, with ultrasound being the primary modality. Computed tomography and magnetic resonance imaging are also widely used [18,19]. Dermoscopic evaluation has pathognomonic features to diagnose the umbilical endometriosis and take fine needle aspiration cytology. However, histopathological analysis remains the primary diagnostic modality to confirm the diagnosis. The histopathology shows a lesion containing ectopic endometrial tissue with chronic inflammation, hemorrhage, and cystic dilatation of the glands [5]. 

The primary treatment of umbilical endometriosis predominantly involves complete surgical excision with a free margin [3,5,18,20]. Recurrence is very low if the lesion is excised completely with primary closure [20]. 

In this report, we describe a 38-year-old married woman with no previous history of pelvic or laparoscopic surgery, aside from a lower-segment cesarean section five years prior. She presented with cyclical symptoms of swelling and pain, consistent with endometriosis. Clinical examination and imaging findings raised suspicion of endometriosis, which was confirmed post-excision through histopathology, revealing ectopic endometrial tissue, hemorrhage, and dilated glands. 

Differential diagnoses were carefully considered. The Sister Mary Joseph nodule, a metastatic nodule primarily from gastric or colonic cancer, was ruled out based on the absence of gastrointestinal and systemic symptoms such as weight loss or lymphadenopathy. Another differential diagnosis is an umbilical hernia. While umbilical endometriosis can occur alongside hernias, hernias may present independently and fluctuate in size due to increased intra-abdominal pressure from chronic cough, constipation, or heavy lifting.

Other differential diagnoses include keloids, papillomas, epidermal cysts, and granulomas secondary to chronic omphalitis or urachal cysts fistulizing to the umbilicus. To distinguish umbilical endometriosis from these conditions, key aspects of the patient's presentation were considered. Endometriosis lesions typically appear as dark red, brown, or blue nodules with a velvety surface and may exhibit cyclical symptoms that vary with the menstrual cycle. In contrast, keloids are raised and shiny, while papillomas appear as wart-like growths with a rough surface. Epidermal cysts are firm, dome-shaped nodules, and granulomas usually manifest as inflamed, reddish lumps. The patient's clinical examination helped eliminate various differential diagnoses, while the histopathological examination confirmed the diagnosis and further distinguished it from other conditions.

## Conclusions

This case demonstrates the critical importance of considering umbilical endometriosis as a potential diagnosis in women presenting with painful umbilical masses. Such considerations are especially relevant for those with a history of abdominal gynecological surgeries or infertility. The presence of ectopic endometrial tissue on the umbilicus can manifest as cyclic pain and swelling, which often correlate with the menstrual cycle. The association is a pathognomonic clinical feature that can aid in the diagnosis of endometriosis and should not be overlooked.

Given the rarity of primary umbilical endometriosis, health care providers may initially misdiagnose the condition, leading to delays in appropriate treatment. Therefore, a high index of suspicion is essential. It is crucial to rule out other potential diagnoses, such as the Sister Mary Joseph nodule, which can present similarly but may indicate underlying malignancy. Misdiagnosing umbilical endometriosis for other conditions could lead to inappropriate management strategies and adversely affect patient outcomes. Complete excision of the tissue is effective in alleviating symptoms and improving patient’s quality of life.
